# Dichroic switching of core–shell plasmonic nanoparticles on reflective surfaces

**DOI:** 10.1002/EXP.20210234

**Published:** 2023-11-20

**Authors:** Tian Liang, Zhiwei Li, Yaocai Bai, Yadong Yin

**Affiliations:** ^1^ Hubei Key Laboratory of Radiation Chemistry and Functional Materials School of Nuclear Technology and Chemistry & Biology Hubei University of Science and Technology Xianning China; ^2^ Department of Chemistry University of California Riverside California USA

**Keywords:** absorbance, dichroic property, localized surface plasmon resonance, plasmonics, reflection, scattering, transmittance

## Abstract

Plasmonic metal nanostructures can simultaneously scatter and absorb light, with resonance wavelength and strength depending on their morphology and composition. This work demonstrates that unique dichroic effects and high‐contrast colour‐switching can be achieved by leveraging the resonant scattering and absorption of light by plasmonic nanostructures and the specular reflection of the resulting transmitted light. Using core/shell nanostructures comprising a metal core and a dielectric shell, we show that their spray coating on reflective substrates produces dichroic films that can display colour switching at different viewing angles. The high‐contrast colour switching, high flexibility in designing multicolour patterns, and convenience for large‐scale production promise their wide range of applications, including anticounterfeiting, mechanochromic sensing, colour display, and printing.

## Graphical abstract

Spray coating of the obtained core/shell nanostructures (a metal core and a dielectric shell) on reflective substrates produces dichroic films that can display colour switching at different viewing angles. The unique dichroic effect and high‐contrast colour‐switching are achieved by leveraging the resonant scattering and absorption of light by plasmonic nanostructures and the specular reflection of the resulting transmitted light.

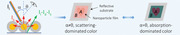

## 1 INTRODUCTION

Plasmonic nanostructures can interact with light through plasmon–photon coupling,^[^ ^1^, ^2^, ^3^, ^4^ ^]^ an effect called the localized surface plasmon resonance (LSPR).^[^ ^5^, ^6^, ^7^ ^]^ Light absorption at a resonant wavelength makes nanostructures of noble metals such as gold and silver display brilliant colours.^[^ ^8^, ^9^, ^10^ ^]^ The LSPR frequency highly depends on size, shape, chemical component, and surrounding dielectrics and can be tuned across a broad spectrum.^[^ ^11^, ^12^, ^13^, ^14^, ^15^, ^16^, ^17^ ^]^ In particular, plasmonic nanostructures with anisotropic shapes feature multiple plasmonic modes, which can be excited depending on their relative orientation to the incident light.^[^ ^18^, ^19^, ^20^, ^21^, ^22^ ^]^ This linear dichroism can generate variable colours corresponding to the resonance modes, which is useful for colour display/presentation and colorimetric sensing.^[^ ^23^, ^24^, ^25^, ^26^ ^]^ Furthermore, coating the plasmonic nanoparticles with a dielectric shell can significantly alter the resonance modes, providing an effective method to tune the plasmonic properties for constructing active optical devices.^[^ ^7^, ^27^, ^28^, ^29^, ^30^, ^31^, ^32^ ^]^ In addition, assembling plasmonic nanoparticles into superstructures induces plasmon coupling between neighbouring nanoparticles, which provides an efficient approach to tuning the resonance wavelength, representing an advanced technique to control plasmonic colour over a broad spectrum.^[^ ^33^, ^34^, ^35^, ^36^ ^]^

Plasmonic coloration has been used in practical applications since ancient times, particularly in windows made of stained glass.^[^ ^36^, ^37^ ^]^ Another typical example is the Lycurgus Cup, which contains gold and silver nanoparticles.^[^ ^38^, ^39^, ^40^ ^]^ It can display two colours when viewed in reflected and transmitted light.^[^ ^41^, ^42^ ^]^ Although the dichroism of the cup is well known, its underlying mechanism has not been actively explored for the development of optical devices.^[^ ^43^, ^44^ ^]^ Efforts using laser printing or nanoimprinting methods to deposit plasmonic nanoparticles on transparent substrates only led to low‐contrast colour‐switching with limited scalability.^[^ ^45^, ^46^, ^47^, ^48^ ^]^ Practical applications of the plasmonic dichroism require developing efficient methods for preparing plasmonic films with an efficient mechanism for balancing scattering and absorption properties.

Herein, we report the development of robust colour‐switching plasmonic films by exploiting the dichroic properties of core–shell plasmonic nanostructures. Specifically, Ag nanoparticles are first coated with a layer of dielectrics or semiconductors (e.g., SiO_2_, Fe_3_O_4_, Cu_2_O, TiO_2_) to produce core/shell nanostructures with minimized interparticle plasmonic coupling and highly tunable coloration in the visible spectrum. These plasmonic nanostructures are printed on reflective substrates as thin films with controllable thicknesses using the readily accessible spray coating method. The light transmitted through the plasmonic film is reflected by the reflective substrate, producing an intense colour complementary to the colour induced by the resonant scattering of the plasmonic nanoparticles observed at non‐reflection angles. Combining the viewing‐angle‐dependent dichroic properties with the simple fabrication method enables fast printing of plasmonic films with highly customizable patterns and pre‐designed high‐contrast colour changes, which may be used for anticounterfeiting, colorimetric sensing, information encryption, and plasmonic colour printing.

## 2 RESULTS AND DISCUSSION

**FIGURE 1:**
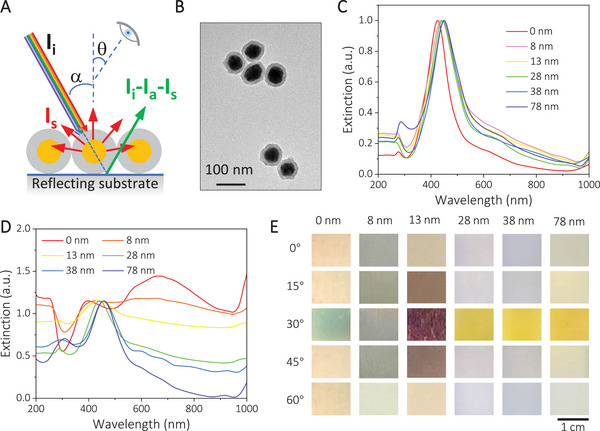
The dichroic property of plasmonic nanoparticles on a reflective substrate. A, Schematic illustration. B, TEM image of typical Ag@SiO_2_ nanoparticles. C, Extinction spectra of an aqueous solution of Ag@SiO_2_ nanoparticles with different silica thicknesses. D, Extinction spectra of thin films of Ag@SiO_2_ nanoparticles of varied silica thicknesses deposited on glass substrates. E, Digital photos of the plasmonic films under different viewing angles (*θ*). The incident angle is 30°.

Ag nanoparticles were prepared as a colloidal dispersion in water using polyvinylpyrrolidone (PVP) as the capping ligand, and then coated with silica of various thicknesses using a sol–gel method.^[^ ^49^ ^]^ The obtained plasmonic nanoparticles were dispersed in an isopropyl alcohol (IPA) solution and sprayed onto a stainless‐steel foil using an airbrush. These plasmonic nanoparticles produce resonant scattering and absorption at their characteristic LSPR positions. As shown in Figure 1A, when the white incident light (*I_i_*) is illuminated on the particles, some light is absorbed (*I_a_*) and some scattered (*I*_s_) at their resonant wavelength. The remaining light (*I_i_—I_a_*—*I_s_*) transmits through the nanoparticle layer and is reflected by the reflective stainless‐steel foil underneath, producing a colour that can be perceived only at the angle of reflection. At other viewing angles, the film's colour results from the resonant scattering (*I_s_*) of plasmonic nanoparticles. Since the resonant scattering is less directional than the specular reflection, the scattered light can be perceived over a wide viewing angle (Figure 1A). In addition, for the spherical plasmonic nanoparticles, the reflection colour is complementary to that caused by resonant scattering. Therefore, the perceived colour of the film can not only be tuned by controlling the resonant properties of the plasmonic nanoparticles but also be switched between the scattering colour and the complementary reflection colour by changing the viewing angle.

The synthesized Ag@SiO_2_ particles feature a core/shell structure. Figure 1B shows the transmission electron microscopy (TEM) image of a typical sample of such particles, comprising Ag cores with an average diameter of 58 nm and SiO_2_ shells with an average shell thickness of 12 nm. With increasing silica thickness from 0 to 78 nm (Figure S1, Supporting Information), the extinction peak of the Ag@SiO_2_ particles redshifted from 425 to 450 nm (Figure 1C) due to the enhanced refractive index surrounding the Ag nanoparticles. They also exhibited different extinction spectra when sprayed onto a glass substrate (Figure 1D). For Ag nanoparticles with only PVP but without SiO_2_ shells, their film showed two peaks in the extinction spectra, with the additional broad peak at ≈673 nm due to strong plasmonic coupling. Coating the Ag nanoparticles with increasing thickness gradually separated the Ag cores and decreased the coupling, nearly diminishing the coupling peak when the SiO_2_ thickness approached 28 nm. Consistent with their dispersion form, the nanoparticle film also exhibited a gradual redshift in the primary resonance peak with increasing silica coating, showing a peak at 457 nm for those with 78‐nm thick silica shells. When the Ag@SiO_2_ nanoparticles were sprayed onto a stainless‐steel foil, these samples displayed viewing angle‐dependent colours. As shown in Figure 1E, except for the one with 8‐nm silica, all the other films exhibited noticeable colour changes when they were viewed near the angle of reflection (*θ* = 30°). A close analysis suggests that all five samples showed complementary colours to their plasmonic resonances at 30°. At other angles, however, the perceived colours were consistent with the films' plasmonic peak positions, suggesting that scattering dominates. This result verifies the proposed design principle of achieving the unique dichroic properties by depositing thin films of the plasmonic nanoparticles, each with a metallic core and a dielectric shell, on reflective surfaces. As for the 8‐nm sample, its plasmonic peak is so broad that it is difficult to distinguish the reflected and scattered light, even by changing the viewing angles. This intriguing observation suggests that a distinct plasmonic peak rather than an overly broad one is required for films to exhibit pronounced dichroic responses.

**FIGURE 2:**
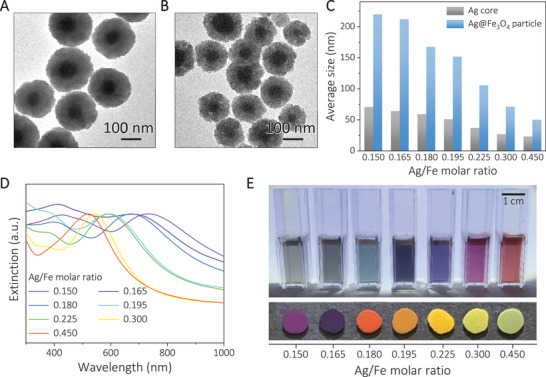
Ag@Fe_3_O_4_ core‐shell nanoparticles with tunable plasmonic properties. A, B, TEM images of Ag@Fe_3_O_4_ nanoparticles with Ag/Fe molar ratios of A, 0.150 and B, 0.180. C, Size distribution, and D, extinction spectra of the Ag@Fe_3_O_4_ nanoparticles prepared with different Ag/Fe molar ratios. E, Digital photos of the Ag@Fe_3_O_4_ samples in colloidal dispersions (top panel) and dry films deposited on a glass slide (bottom panel).

Colour switching can be achieved in a broader spectrum when materials of higher refractive indices than SiO_2_ are used for constructing the shells. For example, we show in Figure 2A,B the successful synthesis of Ag@Fe_3_O_4_ core/shell nanostructures using a polyol reduction process.^[^ ^50^, ^51^ ^]^ The Ag core diameter and the Fe_3_O_4_ shell thickness could be controlled by changing the reaction time or the Ag/Fe molar ratio of the precursor (Figure S2, Supporting Information). While the Ag/Fe ratio of 0.150 produced an Ag core of 71 nm and a Fe_3_O_4_ shell of 75 nm, the core and shell dimensions decreased to 23 and 14 nm at an Ag/Fe ratio of 0.450 (Figure 2C).

When the ratio of Ag/Fe in the particles was increased from 0.150 to 0.450, the main plasmon peaks of the nanoparticles blueshifted from 734 to 520 nm (Figure 2D). A similar blueshift from 664 to 513 nm was observed if these nanoparticles were deposited on glass substrates (Figure S3, Supporting Information). Such manipulation of plasmonic resonance allowed these nanoparticles to exhibit diverse colours in the forms of both aqueous dispersions and solid films deposited on glass substrates (Figure 2E). The aqueous dispersions showed colours consistent with the light absorption of the dispersed nanoparticles at the resonant wavelength. In contrast, the perceived colours of their thick solid films on glass substrates changed from red to green as the Ag/Fe ratio increased from 0.150 to 0.450, which were dominated by plasmonic scattering and, therefore, different from the colours of their corresponding aqueous dispersions.

**FIGURE 3:**
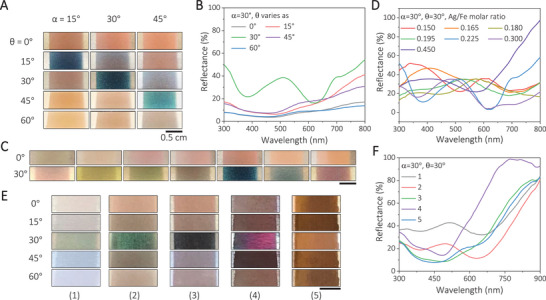
The dichroic properties of Ag@Fe_3_O_4_ plasmonic nanoparticles. A, Digital photos of an Ag@Fe_3_O_4_ nanoparticle film (Ag/Fe molar ratio of 0.225) deposited on a stainless‐steel substrate under different angles of incidence (*α*) and reflection (*θ*). The reflection angle is defined as critical angles at *θ* = *α* and non‐critical angles at *θ* ≠ *α*. B, Reflectance spectra of the sample in A, with *α* = 30°. C, Digital photos of different Ag@Fe_3_O_4_ nanoparticles *θ* = 0° (top) and *θ* = 30° (bottom) while keeping *α* = 30°. From left to right, the films were made from Ag@Fe_3_O_4_ particles with the Ag/Fe atomic ratios of 0.150, 0.165, 0.180, 0.195, 0.225, 0.300, and 0.450. D, The corresponding reflection spectra of different Ag@Fe_3_O_4_ nanoparticles when *α* = *θ* = 30° at the critical angle. E, Digital photos of Ag@Fe_3_O_4_ (Ag/Fe molar ratio of 0.225) films with different thicknesses on stainless‐steel substrates with *α* = 30° and varying *θ*. From left to right, the areal density of Ag atoms is (1) 0.33, (2) 0.83, (3) 1.33, (4) 2.00, and (5) 2.50 μmol cm^−2^. F, The corresponding reflectance spectra of the samples in E, when *α* and *θ* are both 30°. The scale bars in A, C, and E are all 0.5 cm.

These Ag@Fe_3_O_4_ nanoparticles were sprayed on stainless steel surfaces to investigate their angle‐dependent and thickness‐dependent dichroic properties. As shown in Figure 3A, the film made of nanoparticles with Ag/Fe = 0.225 shows a similar reddish colour when the incident angle (*α*) differs from the viewing angles (*θ*), and greenish blue under three different incident angles when *α* = *θ*. Their corresponding reflection spectra confirm the colour observation (Figure 3B). When *α* equals *θ*, the reflectance spectrum shows an obvious peak at 515 nm, which is green. Spectra measured at other angles only show relatively high reflectance at long wavelengths, which is responsible for the reddish colours. Again, this viewing angle‐dependent dichroism can be explained by the scattering and absorption of nanoparticles at the LSPR positions. At critical angles (*α* = *θ*), the specular reflection of the stainless steel substrate is so strong that the transmitted light through the film is reflected again to dominate the perceived colours. As the light propagates through the thin film, the part with the plasmonic resonant wavelength is absorbed by the nanoparticles, only allowing its complementary parts to be observed. In other viewing angles, the resonant scattering dominates due to the relatively weak reflection of the substrates at these non‐critical angles. The working principle was found to be general by studying films of Ag@Fe_3_O_4_ particles with the Ag/Fe ratio from 0.150 to 0.450, which all undergo a significant colour change at critical angles (Figure 3C). They also show obvious peaks in the corresponding reflection spectra when *α* = *θ*, consistent with the perceived colours (Figure 3D). In addition, the first peak of each sample blueshifts from 617 to 410 nm as the Ag/Fe molar ratio increases from 0.150 to 0.450, respectively.

The dichroic property of the film depends on not only the viewing angle but also the film thickness. We used the nanoparticles with the Ag/Fe molar ratio of 0.225 as building blocks to prepare five films with gradually increasing thicknesses. Their thickness difference could be directly observed in the scanning electron microscopy (SEM) images (Figure S4A, Supporting Information). When *α* = 30° and *θ* = 0°, as shown in the first row of Figure 3E, films with different thicknesses exhibited similar brownish colour and reflection spectra (Figure S4B, Supporting Information), albeit at different intensities. However, at the critical angles (*α* = *θ* = 30°), the samples displayed different colours as the thickness of Ag@Fe_3_O_4_ increased from the left to the right (third row in Figure 3E). For the thickest film, the incident light could not transmit through the film and be reflected by the stainless‐steel substrate. Therefore, scattering would dominate even at the critical angle, leading to viewing angle‐independent colours. When the film became thinner, the intensity of reflected light gradually increased, leading to a varying ratio between the transmitted and scattered light and, consequently, the changes of the perceived colours depending on film thickness. The ratio changes between the reflected and scattered light could also be interpreted by the thickness‐dependent reflection spectra of the five samples (Figure 3F). The spectra of thin films showed obvious peaks in the visible spectrum at critical angles, consistent with their green colours. As film thickness increased further, such a peak gradually diminished, suggesting attenuated reflection (Figure S4C, Supporting Information).

**FIGURE 4:**
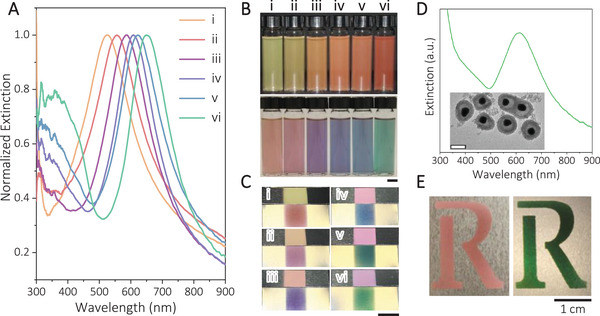
The dichroic properties of other core–shell plasmonic nanoparticles. A, Extinction spectra of the colloidal dispersion of Ag@Cu_2_O@SiO_2_ nanoparticles with increasing Cu_2_O thicknesses: (i) 4 nm, (ii) 6 nm, (iii) 10 nm, (iv) 13 nm, (v) 16 nm, and (vi) 25 nm. B, Digital photos showing aqueous dispersion of Ag@Cu_2_O@SiO_2_ nanoparticles with light illuminating from the front (top) and back (bottom). C, Digital photos of Ag@Cu_2_O@SiO_2_ nanoparticles deposited on stainless‐steel substrates. The top and bottom panels in each group were taken with *α* = 30°, *θ* = 60°, and *α* = *θ* = 30°, respectively. D, Extinction spectra of an aqueous dispersion of Ag@TiO_2_ yolk–shell nanoparticles. The inset is the corresponding TEM image with a scale bar of 100 nm. E, Photos of a colour‐switchable letter by depositing Ag@TiO_2_ yolk‐shell nanoparticles on a stainless‐steel substrate taken at non‐critical (left panel) and critical (right panel) viewing angles. The scale bars in B, C, and E are all 1 cm.

The design principle of the dichroic films is general, allowing the use of various plasmonic core‐shell nanoparticles with different shell compositions and thicknesses to produce the colour contrast desired for specific applications. As an additional example, we coated Ag nanoparticles with Cu_2_O shells of increasing thickness from 4 to 6, 10, 13, 16, and 25 nm by adding more precursors (see experimental details). They were further coated with a layer of silica to enhance chemical stability, producing Ag@Cu_2_O@SiO_2_ nanoparticles with resonance peak redshifting from 523 to 648 nm, depending primarily on the thickness of the Cu_2_O shells (Figure 4A). As shown in the upper row of Figure 4B, the aqueous dispersions of Ag@Cu_2_O@SiO_2_ nanoparticles gradually redshifted from yellow to red with increasing thickness when the light illuminated from the front. Under this lighting condition, the perceived colour is dominated by the scattering of nanoparticles. At the bottom panel of Figure 4B, when light is incident from the back of the sample, the part at the resonant wavelength is absorbed and scattered, allowing the observation of the complementary colour in the front view. These Ag@Cu_2_O@SiO_2_ nanoparticles also exhibited dichroic properties when deposited on stainless‐steel substrates (Figure 4C). When α≠θ, the sample displayed scattering‐dominated colour, while the same sample exhibited the complementary colour when *α* = *θ*. These samples exhibited high‐contrast colour switching thanks to their sharp and highly tunable plasmonic peaks in the visible spectrum.

In another demonstration, we have also synthesized Ag@TiO_2_ yolk‐shell nanostructures which exhibited similar dichroic properties. Figure 4D shows the morphology and extinction spectrum of a typical sample with plasmonic resonance at 610 nm, which is in the red gamut. Such nanoparticles were deposited as the letter “R” on a stainless‐steel substrate (Figure 4E). When *α* ≠*θ* (left panel), the letter appeared red due to scattering‐dominated coloration. At the critical angle (*α* = *θ*, right panel), the letter turned from red to green, consistent with the red‐green complementary colour combination.

**FIGURE 5:**
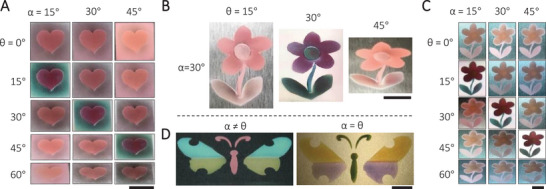
Patterned dichroic films of core‐shell plasmonic nanoparticles. A, Digital photos of a dichroic film of a heart pattern taken at different angles of incidence and observation. The heart pattern and background were made of Ag@Fe_3_O_4_ nanoparticles with the Ag/Fe molar ratio of 0.450 and 0.225, respectively. B, Digital photos of a dichroic flower pattern at 30° incidence and various viewing angles. The flower pattern was fabricated by depositing Ag@Fe_3_O_4_ nanoparticles with Ag/Fe molar ratios of 0.180, 0.225, 0.300, and 0.450 at the stem, leaves, pistil, and petals, respectively. C, Digital photos of a dichroic flower pattern taken at different angles of incidence and observation. The pattern was fabricated by depositing Ag@Fe_3_O_4_ nanoparticles into films of different thicknesses in different areas of the flower. The Ag/Fe molar ratio is 0.225. D, Digital photos of a dichroic butterfly pattern taken at the non‐critical (left) and critical (right) angles. The pattern was fabricated by depositing Ag@SiO_2_ nanoparticles of different silica shell thicknesses in different areas. The scale bars are all 1 cm. All the above patterns were deposited on stainless‐steel substrates.

The availability of various core–shell plasmonic nanoparticles and their unique dichroic property offer many ways to prepare colour‐switching patterns desired for a wide range of applications, such as anticounterfeiting devices and colour displays. For example, we sprayed a heart pattern on a stainless‐steel substrate using the Ag@Fe_3_O_4_ nanoparticles with Ag/Fe molar ratios of 0.450 and then the background using those with the Ag/Fe molar ratio of 0.225 (Figure 5A). At non‐critical angles, there was no high contrast between the pattern and the background because their perceived pinkish colours were dominated by scattering, which was not significantly different from these two types of nanoparticles (Figure 3C). At the critical angle, however, the heart appeared deep red while the background turned green, producing a high contrast due to the reflection‐dominated coloration. When multiple types of plasmonic nanoparticles were used, colour switching of more complex patterns could be achieved. To demonstrate this possibility, we prepared a flower pattern by spraying Ag@Fe_3_O_4_ nanoparticles with Ag/Fe molar ratios of 0.180, 0.225, 0.300, and 0.450 for the stem, leaves, pistil, and petals, respectively, with the area densities of the nanoparticles being controlled at around 1.0 μmol cm^−2^ (based on Ag) for all parts. As shown in Figure 5B, the flower showed drastically different hues when observed at critical and non‐critical angles. In Figure 5C, we further demonstrated that a dichroic flower pattern could be fabricated using the same nanoparticles but different film thicknesses for different parts. Ag@Fe_3_O_4_ nanoparticles with the Ag/Fe molar ratio of 0.225 were sprayed onto a stainless‐steel substrate, producing a flower pattern with nanoparticle densities of 0.5, 1.0, 2.0, and 2.5 μmol cm^−2^ for the stem, leaves, pistil, and petals, respectively (based on Ag). Changing the incidence and viewing angles led to dynamic colour changes due to the thickness‐dependent dichroic property discussed in Figure 3E. As a final demonstration, we sprayed a butterfly pattern using Ag@SiO_2_ nanoparticles with SiO_2_ shells of different thicknesses (Figure 5D). The multicolour pattern exhibited completely different hues when switched between *α*≠*θ* (left) and *α* = *θ* (right).

## 3 CONCLUSION

This work reports the dichroic properties and colour switching of plasmonic nanostructures with different chemical components and morphologies by taking advantage of their unique resonant absorption and scattering at tunable wavelengths. By carefully controlling the peak positions and lighting conditions, we demonstrate vivid colour switching of core/shell and yolk/shell nanostructures in both colloidal solutions and solid films. This interesting dichroic property is general to plasmonic nanostructures with obvious extinction peaks, making it possible to realize coloration and colour switching in the whole visible spectrum. Enabled by this unique optical effect and readily available spray coating method, many colour‐changing patterns are prepared on reflective substrates, which display designated switching between two colours depending on the nanoparticle resonant frequency and film thickness. Robust plasmonic films can therefore be printed with encrypted information or customizable patterns observable only at a specific angle. This unique dichroic property, in combination with the easy coating methods, represents a promising approach to developing colour‐switching devices for anticounterfeiting, product authentication, and multicolour printing.

## AUTHOR CONTRIBUTIONS

Yadong Yin and Yaocai Bai designed this project. Tian Liang, Yaocai Bai, and Zhiwei Li performed the experiments and analyzed the data. The manuscript was written by Tian Liang and Zhiwei Li, and revised by Yadong Yin. All authors have approved the final version of the manuscript.

## CONFLICT OF INTEREST STATEMENT

The authors declare no conflicts of interest.

## Supporting information

Chemical reagents, materials, and synthesis procedures; additional TEM and SEM images of Ag@SiO_2_ and Ag@Fe_3_O_4_ nanoparticles; extinction and reflectance spectra of the Ag@Fe_3_O_4_ nanoparticle films.

## Data Availability

The data that support the findings of this study are available from the corresponding author upon reasonable request.
