# Supplementary material for: Dichroic switching of core–shell plasmonic nanoparticles on reflective surfaces
Source: Exploration (Beijing). 2023 Nov 20;4(3):20210234. doi: 10.1002/EXP.20210234 (PMC11189573; doi:10.1002/EXP.20210234)
Supplement: Supplementary file 1 — Chemical reagents, materials, and synthesis procedures; additional TEM and SEM images of Ag@SiO2 and Ag@Fe3O4 nanoparticles; extinction and reflectance spectra of the Ag@Fe3O4 nanoparticle films. [file EXP2-4-20210234-s001.docx]

Supporting Information

**Dichroic switching of core-shell plasmonic nanoparticles on reflective surfaces**

Tian Liang,^1,2^ Zhiwei Li,^2^ Yaocai Bai,^2^ Yadong Yin^2,^^[[1]](#footnote-1)^

*^1^ Hubei Key Laboratory of Radiation Chemistry and Functional Materials, School of Nuclear Technology and Chemistry & Biology, Hubei University of Science and Technology, Xianning 437100, P. R. China*

*^2^ Department of Chemistry, University of California, Riverside, CA 92521, USA*

Experimental section

### Chemical reagents

Polyvinylpyrrolidone (PVP, Mw=10000), hydrogen tetrachloroaurate(III) trihydrate (HAuCl_4_·3H_2_O, 99%), sodium borohydride (NaBH_4_), acetonitrile, ascorbic acid, silver nitrate (AgNO_3_, 99%), diethylamine (DEA), isopropanol alcohol (IPA), tetraethyl orthosilicate (TEOS, 98%), iron(III) nitrate nonahydrate (Fe(NO_3_)_3_·9H_2_O), ethylene glycol (EG), sodium acetate (CH_3_COONa/NaAc), cupric chloride (CuCl_2_), sodium dodecyl sulfate (SDS), sodium hydroxide (NaOH), hydroxylamine hydrochloride (NH_2_OH·HCl), hydroxypropyl cellulose, titanium tetraethoxide (Ti(OC_2_H_5_)_4_), and hydrofluoric acid (HF) were all purchased from Sigma-Aldrich.

### Synthesis of Au seed and Ag NP

0.25 g PVP (Mw=10000) and 10 μL HAuCl_4_ (0.25 M) were added into 10 mL H_2_O successively. After dissolving, 0.6 mL NaBH_4_ aqueous solution (0.1 M) was then injected under vigorous stirring for 30 s. The solution was aged for 6 h (complete decomposition of NaBH_4_) before serving as the Au seeds.

40 mL PVP (2 g, 5 wt% in H_2_O) aqueous solution, 20 mL acetonitrile, 4 mL ascorbic acid (70.448 mg, 0.1 M) aqueous solution, and 40 mL H_2_O were added into one flask and stirred at 25°C. Then 3 mL AgNO_3_ (50.961 mg, 0.1 M) aqueous solution was added, and 24 μL of the Au seed solution was quickly injected within 30 s. The solution quickly turned from transparent to yellowish and finally dark green within seconds. After stirring for 15 min, the solution was centrifuged and washed with water for 1 time. Then it was centrifuged and dispersed in 20 ml H_2_O for further use.^[1]^ The diameter of the synthesized Ag nanosphere is around 58 nm.

### Synthesis of Ag@SiO_2_

2 ml Ag (30 μmol) aqueous solution and 0.5 ml DEA were added into 40 ml IPA successively in an ice bath. After sonication for 5 min, different amounts of TEOS was added dropwise into the solution. The solution was sonicated for 90 min under 30°C, centrifuged and washed with water and IPA alternatively twice, and dispersed in 10 ml IPA for further use.^[2-3]^

### Synthesis of Ag@Fe_3_O_4_

1.414 g Fe(NO_3_)_3_·9H_2_O, 35 mL ethylene glycol, 2.512 g sodium acetate, and 0.0892 g AgNO_3_ (Ag/Fe molar ratio of 0.150) were transferred to 50 mL Teflon lined stainless-steel autoclave successively and stirred for 30 min. Then the autoclave was put into an oven and heated at 200°C for 4 h. The obtained Ag@Fe_3_O_4_ was rinsed with H_2_O and centrifuged several times.^[4-5]^ The Ag@Fe_3_O_4_ with different particle sizes can be prepared by only changing the reaction time from 1 to 12 h. The particle size can also be tuned by only changing the Ag amount (altering the Ag/Fe molar ratio from 0.150 to 0.450).

### Synthesis of Ag@Cu_2_O@SiO_2_

The core-shell Ag@Cu_2_O was prepared according to the following procedure. 0.4 mL of the prepared Ag NP solution was introduced into 10 mL mixed aqueous solution of CuCl_2_ (0.1 M) and sodium dodecyl sulfate (SDS, 0.0338 M). Afterward, 0.3 mL NaOH aqueous solution (1 M) and 0.5 mL NH_2_OH·HCl aqueous solution (0.2 M) were added and vigorously stirred for 10 s before being aged for 2 h.^[6]^ The obtained Ag@Cu_2_O was centrifuged and further coated with SiO_2_ to synthesize the Ag@Cu_2_O@SiO_2_.

### Synthesis of yolk-shell Ag@TiO_2_

The obtained Ag@SiO_2_ was dispersed in ethanol under stirring. Then hydroxypropyl cellulose and H_2_O were added. At last, titanium tetraethoxide (Ti(OC_2_H_5_)_4_) was added and kept stirring for several hours. The synthesized Ag@SiO_2_@TiO_2_ was centrifuged and immersed in diluted HF to prepare the yolk-shell structured Ag@TiO_2_.^[7-8]^

### Characterization

The morphology and microstructure of the samples were analyzed using the Thermo Fisher Scientific (formerly FEI/Philips) NNS450 scanning electron microscope and Tecnai 12 transmission electron microscope. The extinction spectra and the reflectance spectra of the samples were measured by Ocean Optics HR2000 Spectrometer and Ocean Insight FLAME-T-UV-VIS Miniature Spectrometer.

**Figure S1.** TEM images of Ag@SiO_2_ nanoparticles with different silica thicknesses (from left to right, the thicknesses are 0, 8, 13, 28, 38, and 78 nm in sequence).

**Figure S2**. SEM images of Ag@Fe_3_O_4_ nanoparticles (A) with the same Ag/Fe molar ratio of 0.150 under 200°C for different reaction time from 1 h to 12 h, (B) with different Ag/Fe molar ratios from 0.150 to 0.450 under the same reaction time of 4 h. (C) TEM images of the Ag@Fe_3_O_4_ nanoparticles in the same sequence of Figure S2B.


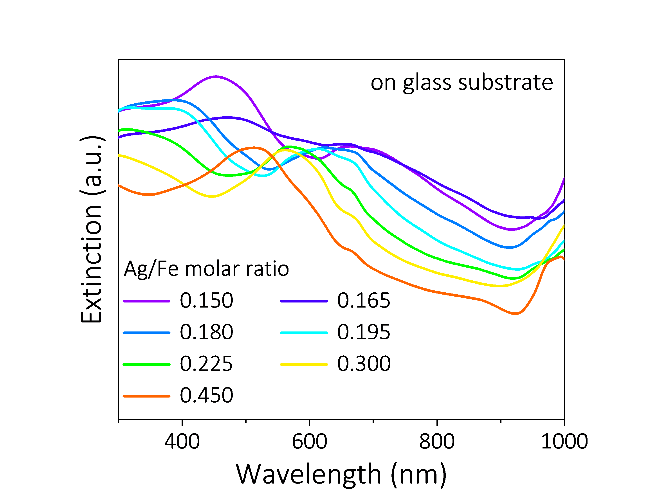


**Figure S3.** Extinction spectra of the Ag@Fe_3_O_4_ nanoparticles with different Ag/Fe molar ratios on a glass substrate.

**Figure S4**. (A) SEM images of Ag@Fe_3_O_4_ particles (Ag/Fe molar ratio of 0.225) with different thicknesses (from 0.33 to 2.50 μmol cm^−2^) on stainless steel substrate. (B) Reflectance spectra of the samples in FigureS4A when α=30° and θ=0°. (C) Intensity of the reflectance spectra measured under α=θ=30° for each thickness of the samples in FigureS4A when the wavelength is fixed at 530 nm and 660 nm.

References

[1] X. Liu, Y. Yin, C. Gao, *Langmuir* **2013**, *29*, 10559.

[2] M. Lismont, C. A. Paez, L. Dreesen, *J. Colloid Interf. Sci.* **2015**, *447*, 40.

[3] O. Niitsoo, A. Couzis, *J. Colloid Interf. Sci.* **2011**, *354*, 887.

[4] V. T. Tran, J. Kim, J. Kim, D. Lee, K.-J. Jeong, J. Lee, *B. Korean Chem. Soc.* **2018**, *39*, 1273.

[5] Y. Zhang, H. Ding, Y. Liu, S. Pan, Y. Luo, G. Li, *J. Mater. Chem.* **2012**, *22*, 10779.

[6] J. Li, S. K. Cushing, J. Bright, F. Meng, T. R. Senty, P. Zheng, A. D. Bristow, N. Wu, *ACS Catalysis* **2012**, *3*, 47.

[7] M. Holgado, A. Cintas, M. Ibisate, C. J. Serna, C. Lopez, F. Meseguer, *J. Colloid Interf. Sci.* **2000**, *229*, 6.

[8] S. Ullah, E. P. Ferreira-Neto, A. A. Pasa, C. C. J. Alcântara, J. J. S. Acuña, S. A. Bilmes, M. L. Martínez Ricci, R. Landers, T. Z. Fermino, U. P. Rodrigues-Filho, *Appl. Catal. B- Environ.* **2015**, *179*, 333.

1. Corresponding author: yadong.yin@ucr.edu (Yadong Yin). [↑](#footnote-ref-1)
